# Bacterial symbiont diversity in Arctic seep *Oligobrachia* siboglinids

**DOI:** 10.1186/s42523-023-00251-x

**Published:** 2023-06-01

**Authors:** Arunima Sen, Gwenn Tanguy, Pierre E. Galand, Ann C. Andersen, Stéphane Hourdez

**Affiliations:** 1 Department of Arctic Biology, The University Centre in Svalbard (UNIS), Longyearbyen, Norway; 2 Faculty of Bioscience and Aquaculture, Nord University, Bodø, Norway; 3 FR2424 Sorbonne Université-CNRS, Genomer, Station Biologique de Roscoff, Roscoff, France; 4 UMR8222 Laboratoire d’Ecogéochimie des Environnements Benthiques (LECOB), CNRS-Sorbonne Université, Observatoire Océanologique, Banyuls-Sur-Mer, France; 5 UMR7144 Laboratoire Adaptation et Diversité en Milieu Marin (AD2M), Sorbonne Université-CNRS, Station Biologique de Roscoff, Roscoff, France

**Keywords:** Frenulates, Chemosynthesis, Methane, Sulfur oxidation, Metabarcoding, Cold seeps

## Abstract

**Background:**

High latitude seeps are dominated by *Oligobrachia* siboglinid worms. Since these worms are often the sole chemosymbiotrophic taxon present (they host chemosynthetic bacteria within the trophosome organ in their trunk region), a key question in the study of high latitude seep ecology has been whether they harbor methanotrophic symbionts. This debate has manifested due to the mismatch between stable carbon isotope signatures of the worms (lower than -50‰ and usually indicative of methanotrophic symbioses) and the lack of molecular or microscopic evidence for methanotrophic symbionts. Two hypotheses have circulated to explain this paradox: (1) the uptake of sediment carbon compounds with depleted δC^13^ values from the seep environment, and (2) a small, but significant and difficult to detect population of methanotrophic symbionts. We conducted 16S rRNA amplicon sequencing of the V3-V4 regions on two species of northern seep *Oligobrachia* (*Oligobrachia webbi* and *Oligobrachia* sp. CPL-clade), from four different high latitude sites, to investigate the latter hypothesis. We also visually checked the worms’ symbiotic bacteria within the symbiont-hosting organ, the trophosome, through transmission electron microscopy.

**Results:**

The vast majority of the obtained reads corresponded to sulfide-oxidizers and only a very small proportion of the reads pertained to methane-oxidizers, which suggests a lack of methanotrophic symbionts. A number of sulfur oxidizing bacterial strains were recovered from the different worms, however, host individuals tended to possess a single strain, or sometimes two closely-related strains. However, strains did not correspond specifically with either of the two *Oligobrachia* species we investigated. Water depth could play a role in determining local sediment bacterial communities that were opportunistically taken up by the worms. Bacteria were abundant in non-trophosome (and thereby symbiont-free) tissue and are likely epibiotic or tube bacterial communities.

**Conclusions:**

The absence of methanotrophic bacterial sequences in the trophosome of Arctic and north Atlantic seep *Oligobrachia* likely indicates a lack of methanotrophic symbionts in these worms, which suggests that nutrition is sulfur-based. This is turn implies that sediment carbon uptake is responsible for the low δ^13^C values of these animals. Furthermore, endosymbiotic partners could be locally determined, and possibly only represent a fraction of all bacterial sequences obtained from tissues of these (and other) species of frenulates.

**Supplementary Information:**

The online version contains supplementary material available at 10.1186/s42523-023-00251-x.

## Background

Bacterial symbionts are essential to siboglinid tubeworms, in fact, as adults, these worms are devoid of a gut, mouth and anus, and all their nutrition is provided by symbionts [20]. Siboglinid larvae, however, do possess a functional transitory digestive tract, and particles have been observed in this gut [41, 83]. In line with this observation, the acquisition of symbionts by siboglinid tubeworms is horizontal: symbionts are not transmitted by gametes, but instead each generation acquires them from the environment where they can be found free-living in the sediment [20]. Adults host symbionts within a specialized structure in the trunk called the trophosome and the morphological modifications associated with symbiont acquisition and the establishment of the trophosome was shown through ultrastructure in the giant hydrothermal vent siboglinid tubeworm *Riftia pachyptila* by Nussbaumer et al. [54]. All hydrothermal vent siboglinids studied to date seem to acquire a single type of symbionts that is shared by all species [50]. In contrast, multiple bacterial strains are hosted by cold seep vestimentiferan siboglinids and which strains make up the symbionts of seep vestimentiferans differ based on geographic region and water depth [50]. Much less is known about the symbionts of smaller, less conspicuous, frenulate siboglinids that are also found across different reducing environments including cold seeps [88].

At high latitude seeps, faunal communities are dominated by frenulate siboglinids of the genus *Oligobrachia* [4, 5, 19, 29, 37, 56, 65, 70, 72–74]. As the primary, and often only confirmed chemosymbiotrophic taxon in these systems, they represent a major contribution to local primary production within these habitats and form the base of the local food chain [6, 18]. However, determining chemosynthetic pathways within Arctic seep frenulates has been a matter of some debate. The first Arctic seep *Oligobrachia* species to be studied was *Oligobrachia webbi* which was formerly known as *Oligobrachia haakonmosbiensis* [75] and was originally named after its location and the first Arctic seep ever investigated, the Håkon Mosby mud volcano (hereafter HMMV) [29, 77]. This species was found to have very low stable carbon isotope values (− 51–56.1‰ in bulk tissue and -70‰ in fatty acids and cholesterol) [29, 48] which is suggestive of methanotrophy [42]. Methanotrophic endosymbionts have been confirmed in another frenulate species, *Siboglinum poseidoni* from the methane-rich Skagerrak seep location in the North Sea [68, 69]. Skagerrak has high concentrations of methane in the sediment [16], similar to the environment at HMMV [17, 25, 53], therefore a methanotrophy-based nutritional symbiosis in *O. webbi* was perfectly plausible. Subsequently, more Arctic and sub-Arctic seep sites were discovered and found to be populated by *O. webbi*, and other *Oligobrachia* species [19, 56, 70, 73, 74, 89], and the methanotrophic symbiont hypothesis was extended and applied to them as well [67, 70, 73].

However, methane is not the only reduced chemical seeping from the subsurface at Arctic seeps; sulfide is also generated in appreciable concentrations in sediment porewater [35, 36, 73] through sediment bacteria that oxidize methane and use sulfate as the final electron acceptor [10, 44]. Sulfide is therefore another possible energy source for Arctic seep *Oligobrachia* worms and indeed, with the exception of *Osedax*, most siboglinids are known to harbor thiotrophic (i.e. sulfide oxidizing) symbionts [20, 26, 33, 86, 87]. Furthermore, genetic approaches failed to detect any methanotrophic symbionts in Arctic seep *Oligobrachia* [48, 70]. Additionally a study using transmission electron microscopy [70] revealed that the morphology of the symbionts of Arctic seep *Oligobrachia* was not consistent with that of known methanotrophic symbionts in other invertebrates [21, 22, 28, 68]. Nonetheless the question of whether Arctic seep *Oligobrachia* host methanotrophic symbionts persisted after the publication of a transmission electron micrograph suggested to be a methanotrophic symbiont in an individual of *Oligobrachia* from a seep in the Laptev Sea [67]. However, there are a few aspects about this image that need to be considered, for example, that only a single instance of this structure was found, the image is highly zoomed-in view of 1–2 bacteria with no overview of the relative abundance of the putative methanotrophs within the tissues, and it is not specified whether it was taken from the trophosome. Furthermore, carbon isotope data was used to corroborate the idea of the presence of methanotrophic symbionts, which as mentioned before, is the confounding variable in determining nutritional modes of these animals. Therefore, Arctic *Oligobrachia* display carbon isotope signatures that could indicate methanotrophic symbionts, but robust evidence for only thiotrophic symbionts currently exists.

There are two, non-exclusive possible explanations for these divergent results: (1) the worms utilize and take up local, isotopically light carbon from the sediment (CO_2_ or organic carbon) from across their epidermis [47, 48] since chemoautotrophy requires an inorganic carbon source, and frenulate worms have been observed to take up organic molecules from the surrounding sediment as well [78–80]. The incorporation of these carbon compounds into worm tissue could account for the highly negative stable carbon isotope values suggestive of methanotrophy [48]. (2) methanotrophic symbionts could be present in Arctic *Oligobrachia* but have just evaded detection with the low throughput cloning/sequencing approaches used so far. In this case, they would likely co-occur with thiotrophic symbionts, but be present in lower proportions. Both sequencing after amplification of the 16S rRNA gene and cloning, and TEM are more biased towards dominant populations and could have simply failed to detect smaller populations of methanotrophic bacteria among the more abundant thiotrophic ones. Whether methanotrophs or sediment carbon uptake is responsible for the mysterious stable carbon isotope values remains an open question, and subsequently, the metabolism of northern latitude seep *Oligobrachia* remains unresolved*.*

**Fig. 1:**
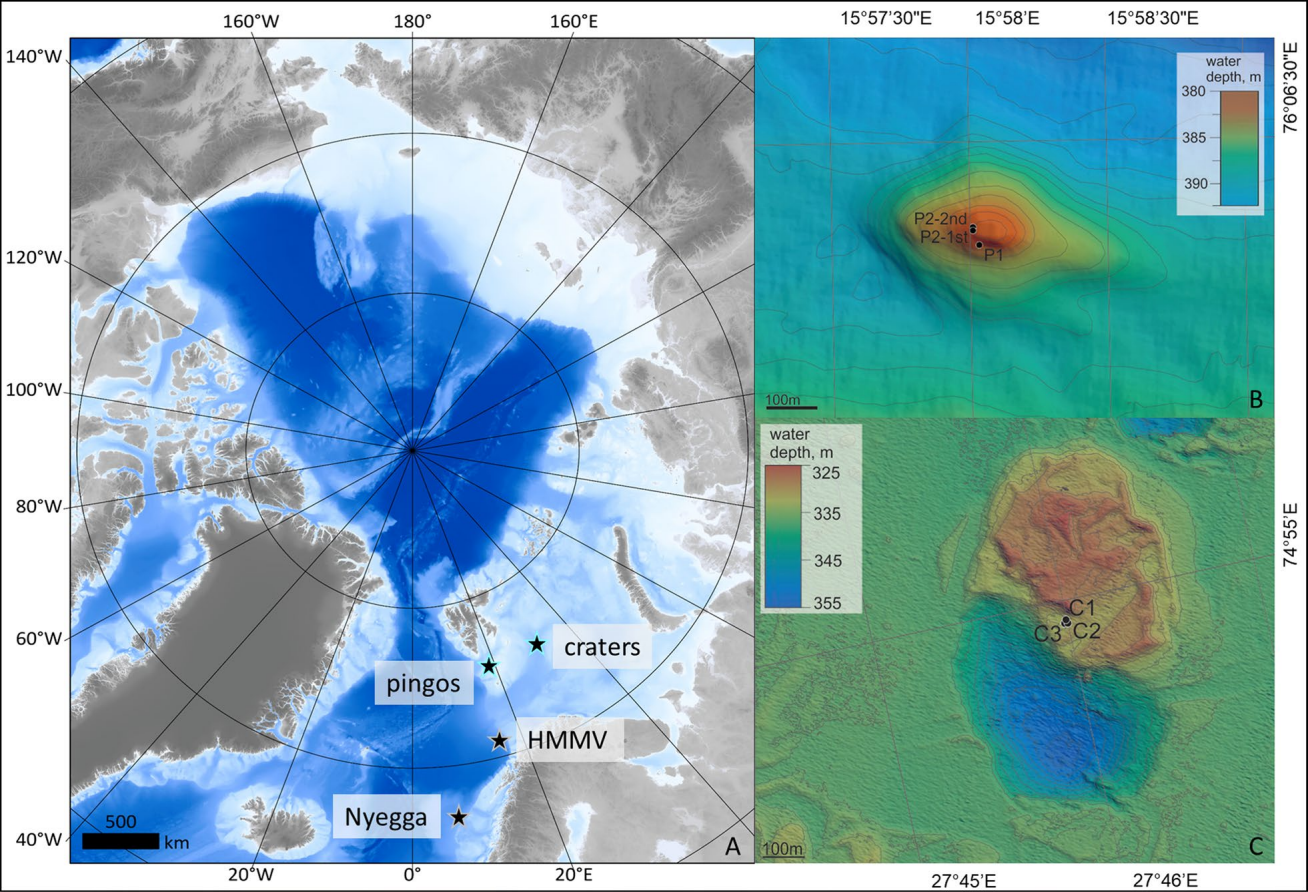
Map of study sites. A: The Arctic with the locations of the sampling location sites marked. Bathymetry was obtained from IBCAO [40]. Stars with gray outlines depict where *O. webbi* is present and was collected from (single collections from the two sites of Nyegga and HMMV). Stars with blue outlines represent locations where *Oligobrachia* sp. CPL-clade is present. The pingo and crater sites are shown in the insets (B and C respectively), with sampling locations (bathymetry from [76] and [2]

**Fig. 2:**
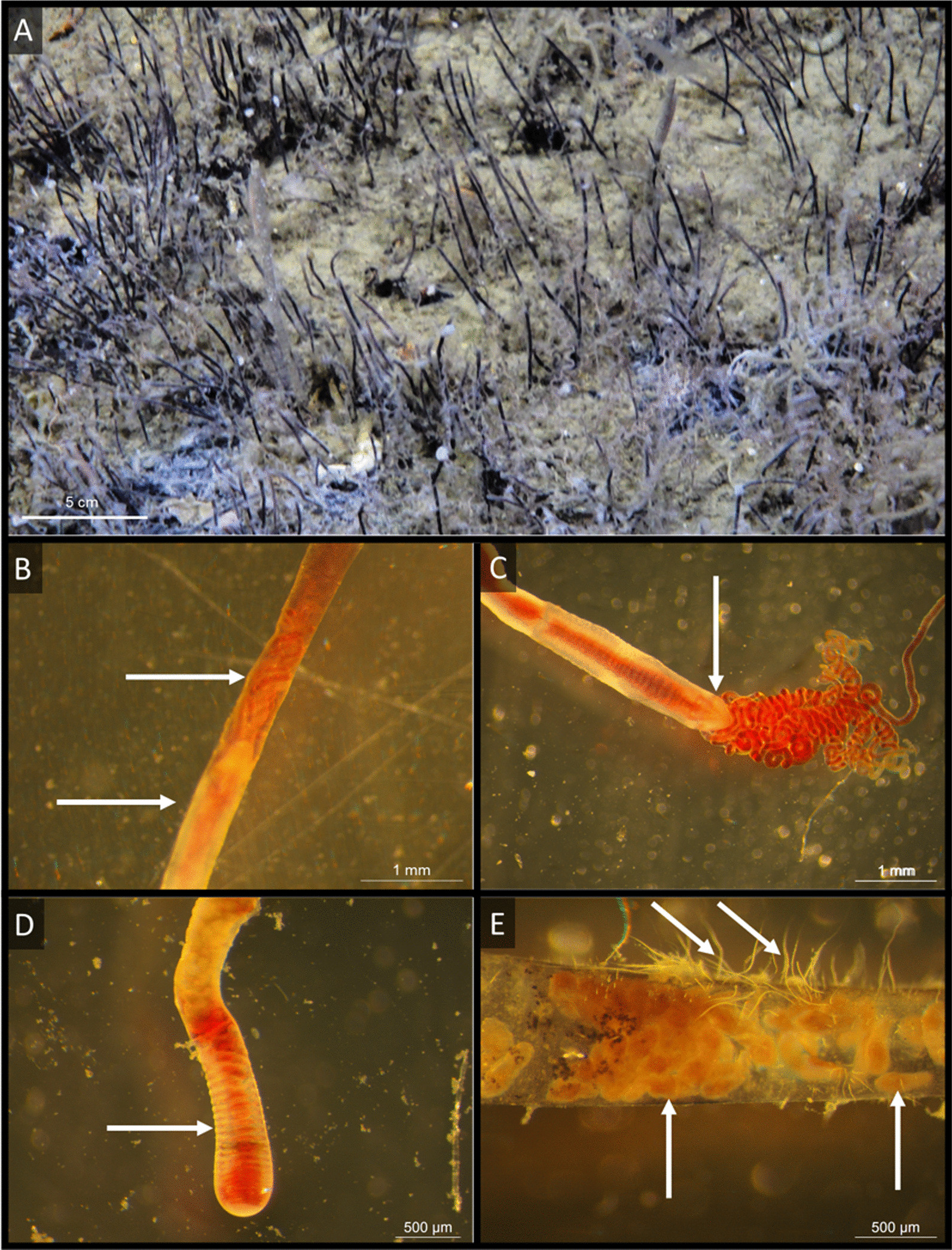
Images of *Oligobrachia* worms and the focus of this study. **A**: In situ image of tufts of *Oligobrachia* worms (image courtesy of the Arctic SGD project). The black hair like extrusions from the sediment surface are the anterior ends of the worms within their brown-black tubes. **B**: The anterior part and tentacles of an *Oligobrachia* sp. CPL-clade worm within its tube. In this part, the tube is translucent and the animal within is visible. Note the pointed head/frenulum (bottom arrow) and the red tentacles extending from it (top arrow). **C**: Anterior of an *Oligobrachia* sp. CPL-clade worm after extraction from its tube, showing several red tentacles attached anteriorly to the pointed frenulum (arrow). **D**: The opisthosome (posterior most end) of an *Oligobrachia* sp. CPL-clade worm extracted from its tube, showing its segmentation (arrow). **E**: Larvae within the tube of an individual worm (upwards pointing bottom arrows). These were present in the transparent, anterior end of the tube, anterior to the worm and its tentacles. On the outside of the tube, note the presence of white, filamentous bacteria (slanted top arrows)

The goal of our study was to test whether high latitude seep *Oligobrachia* contain methanotrophic bacteria, and to overall examine symbiont communities in more detail. We targeted the two most common species found across high latitude seep sites, *O. webbi* and *Oligobrachia* sp. CPL-clade (CPL is an acronym of the colloquial names of the first collection sites for this species), from four different seep sites in the Arctic and sub-Arctic (Nyegga, HMMV, Barents Sea pingo site and Barents Sea crater site, Fig. 1). The four different sites cover differences in latitude, depth and geography, allowing for those factors to additionally be examined with respect to symbiont communities, i.e., verify if there are any patterns of symbiont biogeography. For a precise description of the host-associated bacterial communities, we used high throughput sequencing of the V3-V4 region of the 16S rRNA gene, and targeted the trophosome (symbiotic tissue), as well as the anterior, non-symbiotic part of some of the animals (Fig. 2). We additionally conducted a morphological inspection of the symbiont-containing trophosome organ through electron microscopy for *O. webbi* since this has not been conducted to date on this species (it has only been conducted on *Oligobrachia* sp. CPL-clade, [67, 70].

## Results

### Diversity of bacterial communities

A total of 4 202 004 reads were obtained from the 30 individual samples we processed. After filtering, the total number of reads was reduced to 4 155 465 (about 99% of the total reads). The number of reads per sample ranged from 16 939 for HM3 to 367 244 for C3-2 (Table 1). Letters in the sample names indicate the geographic origin of the specimens (see Methods section).

**Table 1:** Details of the 30 samples used in this study and results of the sequencing efforts for each

Sample name	Sample location (letters in sample name)	Collection and individual (# in sample name)	Tissue type/region	Number of reads	Number of ASVs	Number of families
C1-1	Craters (Bjørnøyrenna)	Collection 1, individual 1	Trophosome	237,289	73	25
C1-2	Craters (Bjørnøyrenna)	Collection 1, individual 2	Trophosome	87,286	64	32
C2-1	Craters (Bjørnøyrenna)	Collection 2, individual 1	Trophosome	227,409	69	22
C2-2	Craters (Bjørnøyrenna)	Collection 2, individual 2	Trophosome	114,577	41	17
C2-3	Craters (Bjørnøyrenna)	Collection 2, individual 3	Trophosome	242,256	44	13
C3-1	Craters (Bjørnøyrenna)	Collection 3, individual 1	Trophosome	106,166	20	6
C3-2	Craters (Bjørnøyrenna)	Collection 3, individual 2	Trophosome	367,244	31	8
C3-3	Craters (Bjørnøyrenna)	Collection 3, individual 3	Trophosome	198,264	62	22
C3-4	Craters (Bjørnøyrenna)	Collection 3, individual 4	Trophosome	47,919	19	3
C3-5	Craters (Bjørnøyrenna)	Collection 3, individual 5	Trophosome	86,725	22	5
C3-6	Craters (Bjørnøyrenna)	Collection 3, individual 6	Trophosome	324,712	39	14
C3-6 h	Craters (Bjørnøyrenna)	Collection 3, individual 6	Host only	93,631	290	74
**HM1a + **	Håkon Mosby mud volcano	**Collection 1, individual 1 + **	Trophosome	18,309	37	19
**HM1b + **	Håkon Mosby mud volcano	**Collection 1, individual 1 + **	Trophosome	30,154	27	15
HM2	Håkon Mosby mud volcano	Collection 1, individual 2	Trophosome	44,128	20	10
HM3	Håkon Mosby mud volcano	Collection 1, individual 3	Trophosome	16,939	17	8
N1	Nyegga slide	Collection 1, individual 1	Trophosome	31,254	138	52
N2a*	Nyegga slide	Collection 1, individual 2*	Trophosome	79,620	684	100
N2b*	Nyegga slide	Collection 1, individual 2*	Trophosome	83,501	693	103
P1-1 h	Pingos (Storfjordrenna)	Collection 1, individual 1	Host only	169,774	119	46
P1-2	Pingos (Storfjordrenna)	Collection 1, individual 2	Trophosome	90,920	49	24
P2-1	Pingos (Storfjordrenna)	Collection 2, individual 1	Trophosome	214,643	21	3
**P2-2 ha¤**	Pingos (Storfjordrenna)	**Collection 2, individual 2¤**	Host only	113,821	574	113
**P2-2hb¤**	Pingos (Storfjordrenna)	**Collection 2, individual 2¤**	Host only	68,898	397	90
P2-3	Pingos (Storfjordrenna)	Collection 2, individual 3	Trophosome	126,074	23	6
P2-4	Pingos (Storfjordrenna)	Collection 2, individual 4	Trophosome	177,998	48	14
P2-5	Pingos (Storfjordrenna)	Collection 2, individual 5	Trophosome	152,994	60	25
P2-6	Pingos (Storfjordrenna)	Collection 2, individual 6	Trophosome	263,563	29	6
P2-7	Pingos (Storfjordrenna)	Collection 2, individual 7	Trophosome	124,190	235	69
P2-8	Pingos (Storfjordrenna)	Collection 2, individual 8	Trophosome	215,207	52	9

Column 1 lists each sample used, with the names that have been used throughout the study. The next three columns explain these names: column 2 is the location from where each sample was obtained. The letters used in the sample names are the first letter of the sample locations. Column 3 refers to the numbers used in the sample names. For the pingo and crater samples where multiple collections were made, the first number is the collection number and the second number is the individual number from the collection. For the Nyegga and HMMV samples where single collections were made, the number in the name refers to the individual number. Column 4 lists the type of tissue: whether samples consisted of trophosome tissue (where symbionts are housed) or non-trophosome, host-only and symbiont free tissue. The last three columns provide details of the results: the number of total reads obtained from each sample, followed by the number of ASVs and finally, the families those reads pertain to per sample. Duplicate samples are highlighted with bold text and symbols (+, *, ¤), and host-only symbiont free tissue samples are underlined

A total of 12 119 Amplicon Sequence Variants (ASVs) for the 30 samples were obtained post processing through the DADA2 pipeline. This number came down to 12 005 after removing singletons (sequences which were only ever obtained a single time). Upon discarding ASVs classified as mitochondria, Archaea, Eukaryota or unclassifiable at the kingdom level, this number was reduced further to 3 295 ASVs, which were classified as belonging to 208 families (Table 1, Additional file 1). Each individual sample contained between 17 ASVs for the least covered sample (HM3) to 684 and 693 ASVs (for N2a and b) (Table 1). 

**Fig. 3:**
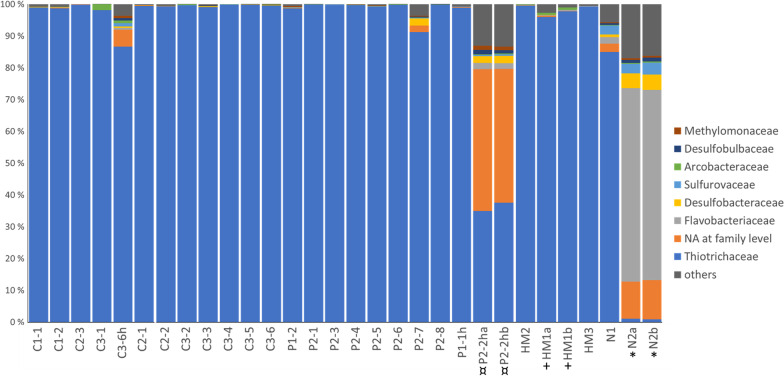
Bar chart of the relative number of reads of different bacterial families in each of the 30 samples used in this study. Duplicate samples are indicated with symbols (+, *, ¤)

Within each individual, the number of families to which ASVs belonged ranged from 3 (for C3-4 and P2-1) to 113 for P2-2 ha (Table 1). Most families, however, were represented by few reads, and the samples were in general dominated by a few families (Fig. 3). The most abundant ASVs by far were classified as Thiotrichaceae, however, there were some other frequent ASVs in some samples from Nyegga that were classified as Flavobacteriaceae (e.g., ASVs 49, 53, 54, 57, 59 and 63, Additional file 1, Fig. 3). A number of ASVs could not be classified at the family level, and among these, ASVs 55, 56 and 58 could not be classified even at the phylum level (Additional file 1). These were present in high frequencies in some of the samples (primarily the Nyegga samples and samples containing some host tissue) (Fig. 3). The most abundant ASV classified as potentially methanotrophic bacteria (ASV 106, Methylmonaceae) was present in only 2 host-only samples (P2-2 h a and b), where it accounted for 0.3% of the total reads (Additional file 1, Fig. 3). Trophosome (i.e. the tissue containing the symbionts) samples did not contain this ASV.

**Fig. 4:**
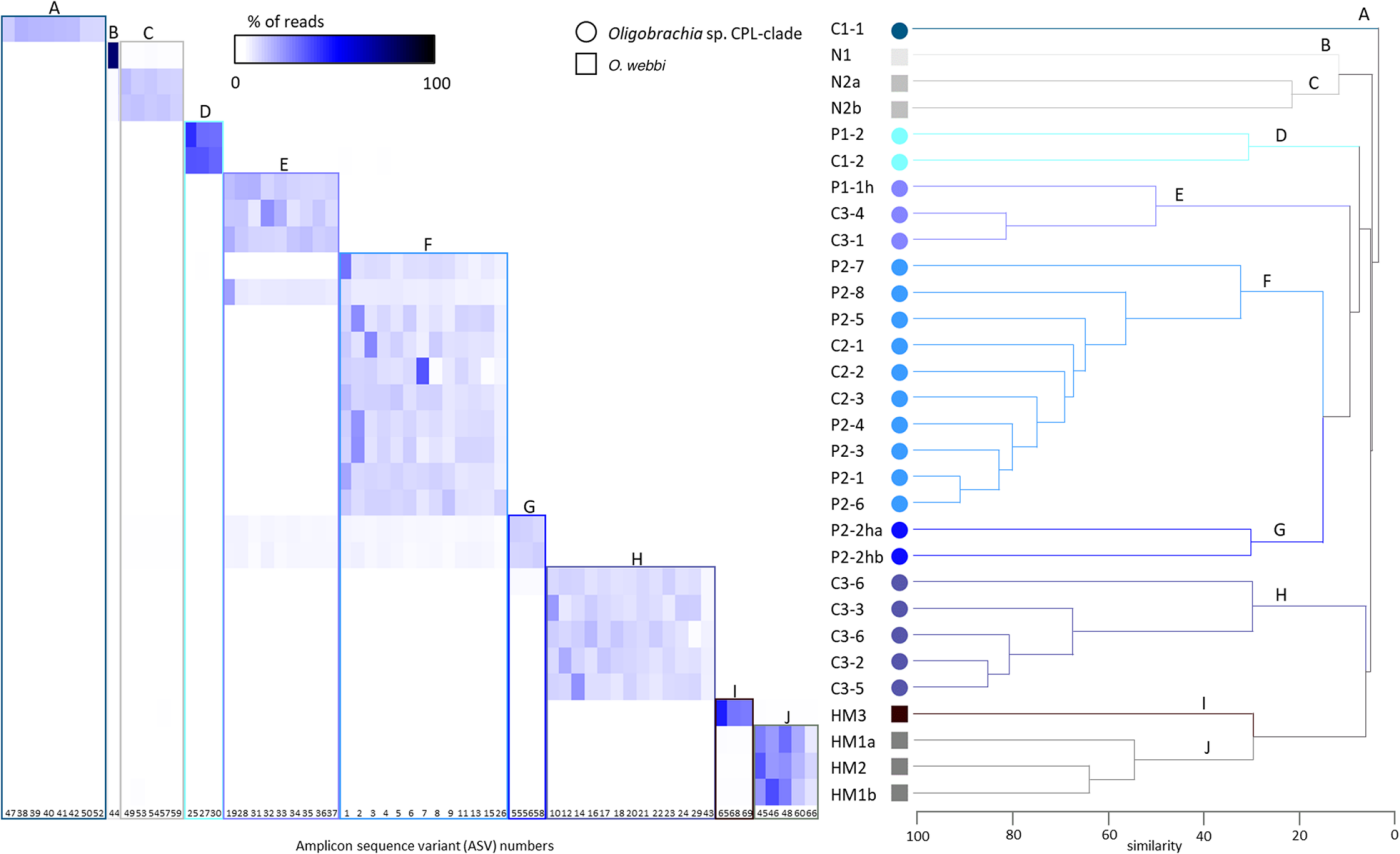
Heatmap of abundances of ASVs (reads) in the samples and dendrogram of the samples based on Bray–Curtis similarity. Groups of ASV combinations present in the samples correspond with the clusters the samples group into, therefore the letters a through J represent both groups of ASV combinations as well as community-based clustering. Shades of gray represent *O. webbi* and shades of blue represent *Oligobrachia* sp. CPL-clade, and specific colors represent community-based clustering as well as the ASV groups (same for both)

Our community analyses (using ASVs) revealed that diversity and evenness indices were consistently higher among samples that contained host tissue (either host only or mixed with symbionts, Table 2). Accordingly, no single ASV was present in these samples in particularly high abundances (at the most 14% of the total reads from individual samples). Other than the N1 sample, no single ASV ever accounted for more than 44% of the reads obtained from the samples (Fig. 4). Therefore, all samples contained multiple dominant ASVs (Fig. 4).

**Table 2:** Diversity and evenness indices of the bacterial communities associated with all the samples in this study, using the ASVs obtained

sample	Richness (S)	Margalef's richness (d)	Pielou's evenness (J')	Fisher's α	Shannon's index (log e) (H')	Simpson's index (1-λ')	Hill index N1	Hill index N2
C1-1	73	5.82	0.52	7.00	2.23	0.88	9.30	8.40
C1-2	64	5.54	0.31	6.76	1.30	0.69	3.68	3.20
C2-1	69	5.51	0.58	6.60	2.46	0.89	11.71	9.21
C2-2	41	3.43	0.59	3.99	2.21	0.84	9.11	6.33
C2-3	44	3.47	0.67	4.00	2.54	0.91	12.66	11.76
C3-1	20	1.64	0.78	1.82	2.33	0.90	10.31	9.56
C3-2	31	2.34	0.73	2.62	2.52	0.91	12.48	11.32
C3-3	62	5.00	0.60	5.95	2.49	0.90	12.11	10.18
C3-4	19	1.67	0.76	1.87	2.22	0.88	9.25	8.02
C3-5	22	1.85	0.79	2.07	2.45	0.90	11.62	9.91
C3-6	39	2.99	0.68	3.40	2.48	0.91	11.91	11.09
C3-6 h	290	25.25	0.58	37.01	3.28	0.94	26.68	16.61
HM1a	37	3.67	0.50	4.45	1.81	0.79	6.10	4.71
HM1b	27	2.52	0.49	2.92	1.63	0.76	5.10	4.12
HM2	20	1.78	0.55	2.00	1.65	0.78	5.19	4.47
HM3	17	1.64	0.44	1.87	1.24	0.67	3.44	2.99
N1	138	13.24	0.28	18.58	1.39	0.39	4.02	1.64
N2a	684	60.52	0.64	102.80	4.16	0.94	64.10	16.71
N2b	693	61.06	0.64	103.52	4.16	0.94	63.77	16.46
P1-1 h	119	9.80	0.50	12.51	2.38	0.89	10.83	9.42
P1-2	49	4.20	0.31	5.00	1.22	0.67	3.39	3.03
P2-1	21	1.63	0.83	1.80	2.52	0.91	12.49	11.11
P2-2 ha	574	49.22	0.69	78.90	4.37	0.96	79.14	27.24
P2-2hb	397	35.55	0.72	55.76	4.31	0.97	74.54	29.15
P2-3	23	1.87	0.78	2.09	2.44	0.90	11.52	9.60
P2-4	48	3.89	0.65	4.54	2.50	0.90	12.18	10.18
P2-5	60	4.94	0.61	5.90	2.48	0.89	11.94	9.52
P2-6	29	2.24	0.76	2.51	2.56	0.92	13.00	12.26
P2-7	235	19.95	0.52	27.98	2.85	0.89	17.32	8.90
P2-8	52	4.15	0.76	4.86	2.99	0.93	19.82	15.09

### Host specific bacterial communities

Samples contained specific combinations of dominant ASVs; these groups of co-occurring ASVs are labeled from A to J in Fig. 4. Each worm was usually host to a single group of ASVs with two exceptions. Sample P2-8 possessed both E and F group sequences, and P2-2 ha and P2-2hb (i*.*e.*,* both replicates of P2-2 h) exhibited mostly group G sequences, but also groups E and F sequences. Each ASV group was found only in one host species or another: the species *Oligobrachia webbi* was host to groups B, C, I, and J, and *Oligobrachia* CPL clade was host to groups A, E, F, G, and H. Though overall, *O. webbi* is host to four groups (B,C, I and J), there appears to be some differentiation based on location, such that Haakon Mosby Mud Volcano animals only hosted groups I and J, while specimens from Nyegga only hosted the B and C groups. In contrast, no such site-based differentiation was seen within *Oligobrachia* CPL clade; all groups of ASVs hosted by this species (A, E, F, G and H) were present in specimens from both the pingo and the crater sites.

Cluster analysis of the samples and their symbiont communities based on Bray–Curtis similarity revealed a number of clusters among the various samples (Fig. 4). Within each cluster, individuals belonged to a single host species, however, host species alone did not determine the clusters: the two clusters containing *O. webbi* were more similar to the CPL-clade clusters than to each other (Fig. 4).

In the clustering we obtained, the samples could be grouped based on the dominant ASVs obtained from them, i.e., the samples grouped into clusters that corresponded exactly with the groups of dominant ASVs they contained (Fig. 4).

### Within- and between-group 16S rRNA sequence divergence

The pairwise Kimura distances between sequences of groups A-J are listed in Table 3. Within each group, the distances ranged from 0 to 0.53% (for groups B and J respectively). Between-group distances displayed very wide ranges: at the lower end, groups H and I displayed differences of 0.54%, and between groups A and E, the calculated distance was 4.09%. Groups C and G (Flavobacteria and unclassifiable at the family level, respectively) were much more divergent, with pairwise distances ranging from 18.71 to 36.51%. Group A stands alone (as do the divergent C and G groups) but groups B, D, and J formed a distinct cluster and groups E, F, H, and I another one. This grouping corresponded with operational taxonomic units (OTUs) based on sequences being at least 97% similar to each other. Therefore, even though the different symbiont groups were found exclusively in one host species or another, OTUs or clusters of symbiont groups were found in both host species. For example, the EFHI cluster was found in both *O. webbi* (group I) and *Oligobrachia* CPL clade (groups E, F, G and H), as was the BDJ cluster (groups B and J in *O. webbi* and group D in *Oligobrachia* CPL clade). The divergent, standalone groups however, were found in only one host species: group C was restricted to *O. webbi* and groups A and G were present only in *Oligobrachia* CPL clade. It should also be noted that not only was group C present only in *O. webbi*, furthermore, it was only present in Nyegga samples, i.e., it was absent in samples from HMMV. The divergent groups in the CPL clade also displayed restrictions in their presence among samples: group A was only present in one individual from the crater site, and group G was only present in the two replicate host tissue samples from a pingo site individual.

**Table 3:** Pairwise average Kimura distances and standard deviation for these distances within (diagonal and italic cells) and between clusters of V3-V4 sequences

	N	Group A	Group B	Group C	Group D	Group E	Group F	Group G	Group H	Group I	Group J
Group A	8	*0.0035* ± *0.0011*									
Group B	1	0.0274 ± 0.0017	*0.0000*								
Group C	5	**0.2792 ± 0.0026**	**0.2748 ± 0.0017**	*0.0041* ± *0.0019*							
Group D	3	0.0252 ± 0.0014	0.0094 ± 0.0025	**0.2696 ± 0.0030**	*0.0036* ± *0.0012*						
Group E	9	0.0409 ± 0.0017	0.0167 ± 0.0019	**0.2794 ± 0.0026**	0.0201 ± 0.0020	*0.0038* ± *0.0014*					
Group F	13	0.0390 ± 0.0018	0.0191 ± 0.0018	**0.2761 ± 0.0025**	0.0171 ± 0.0019	0.0059 ± 0.0018	*0.0041* ± *0.0014*				
Group G	3	**0.2047 ± 0.0019**	**0.1917 ± 0.0016**	**0.3651 ± 0.0017**	**0.1973 ± 0.0014**	**0.1908 ± 0.0019**	**0.1937 ± 0.0018**	*0.0022* ± *0.0000*			
Group H	13	0.0394 ± 0.0019	0.0157 ± 0.0016	**0.2753 ± 0.0025**	0.0172 ± 0.0019	0.0062 ± 0.0019	0.0084 ± 0.0018	**0.1884 ± 0.0020**	*0.0040* ± *0.0015*		
Group I	3	0.0364 ± 0.0019	0.0174 ± 0.0022	**0.2746 ± 0.0020**	0.0145 ± 0.0016	0.0077 ± 0.0018	0.0099 ± 0.0018	**0.1871 ± 0.0020**	0.0054 ± 0.0016	*0.0036* ± *0.0012*	
Group J	5	0.0275 ± 0.0017	0.0087 ± 0.0015	**0.2726 ± 0.0018**	0.0065 ± 0.0014	0.0164 ± 0.0017	0.0186 ± 0.0016	**0.1925 ± 0.0016**	0.0144 ± 0.0018	0.0114 ± 0.0017	*0.0053* ± *0.0038*

Alignment length = 463 bp. N = number of sequences in each cluster. Numbers in bold are values greater than 5%. Group designations are the same as in Fig. 4

Within a species, *O. webbi* hosted only one of the divergent groups (group C), and without this group, divergence ranged from 0.87 (between groups B and J) to 1.74% (between groups B and I). *Oligobrachia* CPL clade however, contained two divergent groups (A and G), as well as groups that were not very divergent (E, F and H), therefore overall, within this species, the range of divergence was much wider. Essentially, within *Oligobrachia* CPL-clade three levels of divergence could be distinguished: (1) Group A, which was divergent from all groups (divergence values ranged from 2.52% with group D to 4.09% with group E), (2) Group D which was also divergent from all groups (divergence values ranged from 1.71% with group F to 2.52% with group A) and (3) groups E, F, and H which were not very divergent from each other (divergence values ranged from 0.59% between groups E and F to 0.84% between groups F and H).

### Similarity with other symbionts from cold seeps

Pairwise distances between sequences were also calculated for symbionts from a number of selected species from cold-seeps (Table 4). Groups B, D, and J were most closely related to one *Oligobrachia* CPL clade symbiont sequence published earlier [71] (less than 1% divergence with accession number MH619700, also from the pingo site). The EFHI group most closely matched a series of sequences from *Oligobrachia* CPL clade from the pingo and crater sites (divergence values ranging from 0.40 to 1.31%; Table 5), as well as from the same host species in the Beaufort Sea, although divergence values cannot be reported for the consensus sequence published [46]. The divergent groups C (Flavobacteria) and G (unclassified) did not match any published symbiont sequences, including the divergent sequence reported in Sen et al. [70–72]. Group A, found in a single *Oligobrachia* CPL clade individual from the crater site, showed only moderate similarity with the two published sequences from *O. webbi* (3.82 and 4.55%) and with symbiont sequences published earlier for *Oligobrachia* CPL clade (2.91% with accession number MH619700). Symbionts from *Siboglinum fjordicum* in Norway were at least 5.38% divergent, but sequences from *Spirobrachia tripeira* from Spain (Gulf of Cadiz) were more similar (3.14% divergence). In fact, the symbionts of *Spirobrachia tripeira* were on average relatively closely related to all groups other than the divergent C and G groups, with pairwise distances ranging from 0.65 to 1.46% with sequences of the BDJ groups and from 1.8 to 2.18 with sequences from the EFHI groups. The symbionts from the Gulf of Mexico vestimentiferan siboglinid *Lamellibrachia luymesi* were less closely related, with pairwise distance values of at least about 10% for all groups. For *Sclerolinum contortum* from the Gulf of Mexico and Håkon Mosby Mud Volcano, the pairwise distances were all greater than 11% (Table 5). For symbionts from *Oligobrachia mashikoi*, from Tsukumo Bay in Japan, pairwise distances were usually greater than 7%, and the lowest value was 5.42% with accession number AB252051 (Table 5).

**Table 4:** Average pairwise Kimura distances (in % ± standard deviation) for the groups identified in this paper and sequences published earlier for the same species (*Oligobrachia* CPL clade, *Oligobrachia webbi*) and other species from the same region (*Siboglinum fjordicum*) and cold seeps from the Gulf of Mexico (*Lamellibrachia luymesi*) and the Gulf of Cadiz (*Spirobrachia tripeira*). Accession numbers (#) of published sequences are listed for the readers’ reference

	*Oligobrachia* CPL clade					*O. webbi*		*S. fjordicum*	*L. luymesi*	*S. tripeira*
Location	Crater	Pingo and crater	Crater	Pingo	Pingo	Håkon Mosby Mud Volcano		Skoge Inlet, Norway	Gulf of Mexico	Gulf of Cadiz
Accession #	MH619696-MH619698	MH619692- MH619695	MH619699	MH619700	MH619701	AM883179	AM883178	EU086766	HE983340	FR682105
Group A	4.07 ± 0.19	4.07 ± 0.19	**27.62 ± 0.26**	2.91 ± 0.19	4.30 ± 0. 20	4.55 ± 0.20	3.82 ± 0.19	5.38 ± 0.17	11.31 ± 0.19	3.14 ± 0.19
Group B	1.54	1.99	**26.66**	1.31	2.21	2.45	2.21	6.15	10.14	0.65
Group C	**30.12 ± 0.28**	**30.12 ± 0.28**	**27.51 ± 0.16**	**29.79 ± 0.28**	**30.45 ± 0.29**	**30.78 ± 0.29**	**30.12 ± 0.28**	**32.49 ± 0.17**	**35.85 ± 0.18**	**30.12 ± 0.28**
Group D	1.91 ± 0.13	1.91 ± 0.13	**27.19 ± 0. 22**	0. 80 ± 0.13	2.14 ± 0.13	2.37 ± 0.13	2.59 ± 0.13	6.77 ± 0.14	10.55 ± 0.15	1.46 ± 0.13
Group E	0. 68 ± 0.18	0. 68 ± 0.18	**26.43 ± 0.25**	2.24 ± 0.19	0. 90 ± 0.18	1.57 ± 0.19	2.24 ± 0.19	6.31 ± 0.17	10.00 ± 0.18	2.02 ± 0.19
Group F	0. 84 ± 0.20	0. 40 ± 0.20	**26.65 ± 0.31**	1.95 ± 0.20	0. 62 ± 0.20	1.73 ± 0.20	2.40 ± 0.20	6.48 ± 0.21	10.18 ± 0.22	2.18 ± 0.20
Group G	**19.51 ± 0.17**	**20.07 ± 0.17**	**29.73 ± 0.21**	**20.77 ± 0.17**	**19.79 ± 0.17**	**19.89 ± 0.17**	**20.20 ± 0.17**	**21.59 ± 0.18**	**18.31 ± 0.17**	**20.20 ± 0.17**
Group H	0. 48 ± 0.50	0. 89 ± 0.42	**25.99 ± 0.49**	2.03 ± 0.46	1.11 ± 0.42	1.36 ± 0.37	2.03 ± 0.25	6.00 ± 0.33	9.67 ± 0.36	1.81 ± 0.21
Group I	0. 65 ± 0.22	1.09 ± 0.22	**25.93 ± 0.21**	1.76 ± 0.22	1.31 ± 0.22	1.09 ± 0.22	2.21 ± 0.22	6.21 ± 0.14	9.64 ± 0.15	1.99 ± 0.22
Group J	1.50 ± 0.10	1.94 ± 0.10	**26.85 ± 0.16**	0. 83 ± 0.10	2.16 ± 0.10	1.95 ± 0.10	2.16 ± 0.10	6.56 ± 0.11	10.32 ± 0.12	1.05 ± 0.10

Numbers in bold are values greater than 15%. Alignment length = 463 bp. Accesion number ranges indicate sequences with identical for the region used

**Table 5:** Pairwise Kimura distances in % and standard deviations between groups A-J and published symbiont sequences from *Oligobrachia mashikoi* and *Sclerolinum contortum*. Accession numbers (#) of published sequences are listed for the readers’ reference

	*O. mashikoi*								*S. contortum*
Location	Tsukumo Bay, Japan								HMMV, Gulf of Mexico
Accession #	AB271125	AB271124	AB271123	AB271122	AB271121	AB271120	AB252051	AB057751	AM883183 HE614013
Group A	7.38 ± 0.17	7.12 ± 0.18	7.14 ± 0.18	7.37 ± 0.18	8.61 ± 0.18	7.60 ± 0.18	5.42 ± 0.17	8.39 ± 0.18	11.35 ± 0.19
Group B	7.45	7.19	7.21	7.97	8.68	7.67	5.95	8.46	10.92
Group C	30.23 ± 0.16	30.78 ± 0.16	31.24 ± 0.17	30.06 ± 0.16	32.32 ± 0.17	29.35 ± 0.16	29.56 ± 0.16	30.05 ± 0.16	35.28 ± 0.18
Group D	7.85 ± 0.15	7.59 ± 0.15	7.61 ± 0.15	8.38 ± 0.15	9.10 ± 0.15	8.07 ± 0.15	6.35 ± 0.14	8.86 0.15	11.34 ± 0.16
Group E	7.86 ± 0.18	8.62 ± 0.18	7.62 ± 0.18	8.39 ± 0.18	9.10 ± 0.18	9.12 ± 0.18	7.36 ± 0.18	8.88 ± 0.18	11.86 ± 0.19
Group F	8.04 ± 0.22	8.80 ± 0.22	7.79 ± 0.22	8.56 ± 0.22	9.28 ± 0.22	9.29 ± 0.22	7.53 ± 0.22	9.05 ± 0.22	12.03 ± 0.23
Group G	20.42 ± 0.17	20.49 ± 0.17	20.14 ± 0.17	20.81 ± 0.18	20.17 ± 0.17	19.86 ± 0.17	20.56 ± 0.17	21.65 ± 0.18	20.95 ± 0.17
Group H	7.54 ± 0.25	8.30 ± 0.36	7.30 ± 0.25	8.06 ± 0.25	8.77 ± 0.26	8.79 ± 0.37	7.04 ± 0.36	8.55 ± 0.26	11.52 ± 0.27
Group I	7.51 ± 0.15	8.27 ± 0.15	7.27 ± 0.15	8.03 ± 0.15	8.74 ± 0.15	8.76 ± 0.15	7.01 ± 0.14	8.52 ± 0.15	11.48 ± 0.16
Group J	7.63 ± 0.11	7.36 ± 0.11	7.39 ± 0.11	8.15 0.11	8.86 ± 0.11	7.85 ± 0.11	6.13 ± 0.11	8.64 ± 0.11	11.10 ± 0.12

Alignment length = 463 bp. Multiple accession numbers indicate sequences with identical for the region used. HMMV = Håkon Mosby Mud Volcano

### Transmission electron microscopy of symbiotic tissue

**Fig. 5:**
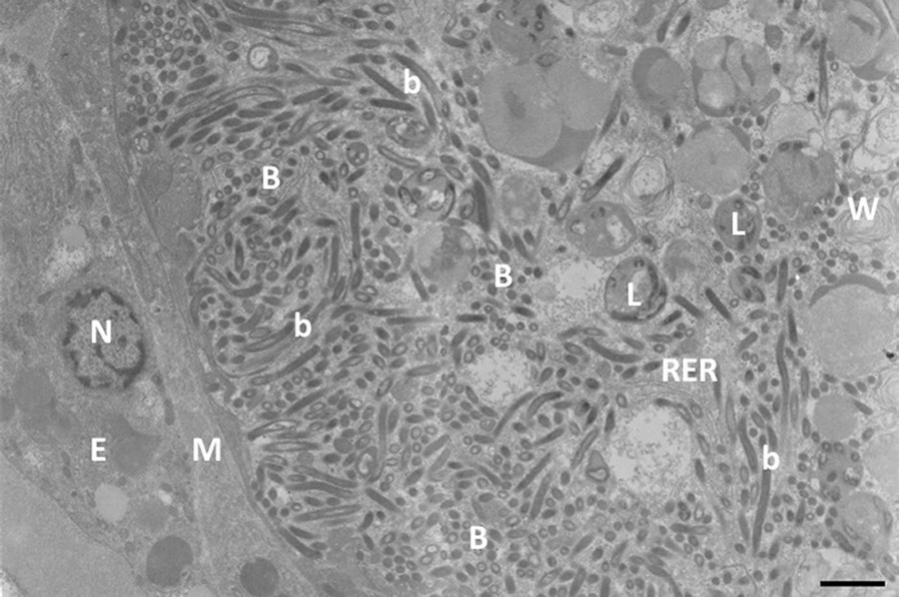
Photomontage of two consecutive transmission electron images of the posterior trophosome of *Oligobrachia webbi* specimen Vi-005 collected at Nyegga showing bacteria with morphology typical of sulfide oxidizing (SOX) bacteria filling the trophosome. B = SOX-bacteria cut in cross-section, b = SOX-bacteria cut longitudinally, E = trunk epidermis, L = lysosomes containing degraded bacteria, M = myoepithelium, N = nucleus, RER = rough endoplasmic reticulum, W = degraded "onion-peel-like" membrane whirl. Scale bar = 2 µm

In all TEM images, we observed elongated cylindrical rod-shaped bacterial symbionts (up to about 3–4 µm long and 0.5 µm in diameter) with clear cytoplasm (Fig. 5). The symbionts were densely packed side by side at the periphery of the trophosome. Towards the center of the body, large vacuoles appeared to contain degraded bacterial symbionts, likely corresponding to lysosomes digesting symbionts. Some of them also contained thin membrane ‘onion-peel-like’ whirls, characteristic of such degradation. However, on all 28 grids we observed from the trophosome tissue, the symbionts contained neither the stacking membranes (characteristic of type I methanotrophs), nor regularly arranged circular peripheral membranes (characteristic of type II methanotrophs).

## Discussion

### Absence of methanotrophic symbionts in Arctic *Oligobrachia*

One of the major motivations for carrying out this study was to attempt to resolve the debate around the nutritional symbiosis and thereby energy sources, of high latitude seep *Oligobrachia*. We chose an extensive and rigorous sequencing methodology with the express aim of uncovering relatively small populations of bacterial endosymbionts that might have been overlooked in past studies and methods. We expected this strategy to inform us whether a difficult-to-discern population of methanotrophic bacteria within Arctic seep *Oligobrachia* worms are responsible for skewing the stable carbon isotope values of these animals away from what one would expect with what appears to be a thiotrophic symbiosis.

Only 100 (out of 3295) ASVs we obtained were classified as methanotrophic bacteria (Methylococcales), and these were represented by very few reads (at most, 0.3% of the total reads within individual samples). Of these, only 21 were obtained from trophosome samples (where they accounted for no more than 0.2% of the total reads). Half of the trophosome-only samples contained no reads whatsoever corresponding to methanotrophic bacteria. This trend, of the few reads of ASVs pertaining to methanotrophic bacteria being skewed towards host-only tissue, is clearly demonstrated by the C3-6 individual: the host-only tissue sample from this individual (C3-6 h) contained 13 ASVs classified as Methylococcales, while the trophosome tissue (sample C3-6) contained a single such ASV (where it accounted for only 0.02% of the total reads). The closest matches to the ASVs classified as Methylococcales within the NCBI database were sequences from deep-sea microbial mats, from epibionts of seep crustaceans, and from sediment samples from hydrothermal vents and seeps [14, 63, 64, 91–93]. One ASV (number 123), which was obtained from a single sample, P2-7, was a 99.57% match for methanotrophic endosymbionts of Bathymodiolus mussels from mid-Atlantic hydrothermal vents, but this was the only ASV that was similar to known methanotrophic endosymbionts, and this ASV was equally similar to gut epibionts of *Rimicaris* shrimp from mid-Atlantic vents [23]. The absence or very low number of reads of ASVs belonging to methanotrophic bacteria from most trophosome samples, and obtained sequences being similar to epibiotic or environmental methanotrophs together suggest that our specimens do not contain endosymbiotic methanotrophs.

Our TEM images did not reveal the presence of bacteria resembling methanotrophic bacteria either, instead, bacteria with morphology typical of sulfur oxidizing bacteria were abundant (Fig. 5). This is the first study to comprehensively sequence *O. webbi* associated bacteria and to visualize the ultrastructure of trophosomal symbionts. Our lack of evidence for methanotrophic symbionts, aligns with similar studies on the CPL-clade [46, 70], as well as fluorescent in situ hybridization assays on both *O. webbi* and the CPL-clade [48, 70]. Therefore, multiple studies and approaches concur that high latitude Arctic *Oligobrachia* appear to lack a methanotrophy-based nutritional mode. The one exception is the structure published by Savvichev et al. [67]. If we assume these authors’ interpretation of this feature is correct in representing methanotrophic symbionts, then the Laptev Sea seep site from which the CPL-clade worms were examined for the study would be an exception to what otherwise seems to be a general pattern of a lack of methanotrophic symbionts in Arctic seep *Oligobrachia.*

### *Oligobrachia* associated bacteria

The pairwise distances within sequence groups were typical of within-genome divergence between copies of the ribosomal genes [85]. Each of the sequence groups therefore likely corresponds to a single genome rather than a consortium of closely-related bacteria. Other than the E and F groups together (which are very closely related) and the host-only P2-2 sample whose sequences do not represent symbiont communities, samples contained one dominant sequence group, indicating that individual *Oligobrachia* worms from high latitude seeps typically host a single dominant bacterial type.

Different individuals though, house different sequence groups in both of the host species we investigated. However, most bacterial sequences from the different sequence groups were very similar to each other, with pairwise distance values at the most being 4.09% for this variable region of the 16S gene. Therefore, the two studied species overall appear to host highly similar groups of bacteria. Two exceptions arose: (1) the C type, found in a single specimen from Nyegga, was very divergent and corresponded to Flavobacteriaceae, and (2) the G type, which was the dominant sequence group in the P2-2 samples, was unclassifiable at the family level. As mentioned earlier, this particular Nyegga sample was a mixture of symbiont-free and trophosome tissue, so whether the C group represents symbiotic partners is questionable, and the P2-2 sample consisted of symbiont-free tissue, therefore, it is unlikely that G type sequences represent symbionts.

We consider groups A, BDJ and EFHI putative symbiont groups since we obtained large numbers of reads from trophosome samples for these groups. Despite the overall similarities across these groups, they do nonetheless represent three different clusters, or even OTUs based on 97% sequence similarity (on full-length 16S sequences). The CPL-clade contained all three clusters/OTUs, whereas *O. webbi* contained the BDJ and EFHI clusters/OTUs. The two latter groups (BDJ and EFHI) are both most closely related to sulfide oxidizers published from *Oligobrachia* CPL clade worms (accession numbers MH619692-MH619695) [70]. Sequences from group A were more divergent than those from clusters BDJ and EFHI but remained relatively close (2.91% divergence with the pingo CPL clade sequence accession number MH619700). It was also relatively close to sequences obtained from another siboglinid species, *Spirobrachia tripeira* symbionts from the Gulf of Cadiz (accession number FR682105). In short, the CPL-clade and *O. webbi* appear to host essentially three types of closely related sulfur oxidizing bacteria.

### Solving the isotope enigma of Arctic *Oligobrachia*

A lack of methanotrophic symbionts means that the very negative stable carbon isotope values of *O. webbi* and the CPL-clade cannot be explained by a ‘hidden’ population of methanotrophic bacteria as has been posited before. The explanation for depleted δC^13^ values then is likely either sediment DOC (dissolved organic carbon) or DIC (dissolved inorganic carbon) uptake by the worms, as suggested by Lösekann et al. [48]. Evidence for the former exists in the form of observations of frenulate siboglinids taking up organic molecules from the sediment [78–80], however, a complicating factor is that frenulates, like all siboglinids, lack digestive organs. Heterotrophic bacterial sequences have been amplified from Gulf of Cadiz seep frenulates [62], therefore, it is possible that heterotrophic endosymbionts aid the animals in processing organic matter they take up from their surroundings. Two of our samples, N2a and N2b contained appreciable numbers of sequences classified as Flavobacteria (Group C) and small numbers of reads of these sequences were also recovered from the P2-2 h sample (Fig. 3). One could suppose that these represent heterotrophic symbionts. However, the Nyegga samples were combined host and trophosome tissue, and the P2-2 h sample was symbiont-free tissue. The closest matches we could find within the NCBI BLAST database to these Flavobacteria sequences are from seep and vent environmental bacteria or microbial mats or epibiotic (but not necessarily symbiotic) bacteria [3, 30, 90, 94]. The restriction, by and large, of these sequences to either host-only tissue or samples containing both host and trophosome tissue, and due to their closest matches being with non-symbiotic bacteria, we suggest that these sequences are from bacteria that are epibiotic or environmental and not endosymbiotic. Group G ASVs could not be classified, and the closest match within the NCBI database of these sequences are to bacteria associated with frenulates from the Gulf of Mexico [62]. However, these sequences were only dominant in the two replicates of host-only tissue samples, therefore it is questionable whether they represent symbiotic bacteria. With this cautious outlook, it seems that, similar to methanotrophic bacteria, the two *Oligobrachia* species studied here do not host heterotrophic endosymbionts.

Uptake of isotopically light sediment DIC is subsequently the most likely explanation for the very negative carbon isotope signatures of high latitude seep *Oligobrachia*. As with any autotrophic organism, *Oligobrachia* requires an inorganic carbon source. At seeps, this can be in the form of either carbon dioxide from the overlying water column, or bicarbonate ions in the sediment generated through the anaerobic oxidation of methane (AOM) coupled to sulfate reduction [10, 44]. Since the source of bicarbonate in sediments at the target sites is isotopically light methane [34, 38, 47], the usage of bicarbonate would be reflected in much more negative isotopic signatures than if seawater carbon dioxide constituted the inorganic carbon source of the worms. Variable carbon isotope values have been measured for *Oligobrachia* across different sites; between -51 and 66.7‰ for *O. webbi* [18, 29, 48] and between − 38.3 and − 57.1‰ for the CPL clade [6, 45, 56]. This is likely indicative of locally available bicarbonate uptake sourced from different, site specific, end-member fluids.

In short, our findings of a lack of methanotrophic bacteria as well as a lack of heterotrophic bacteria, in conjunction with previously reported variable carbon isotope values of worms across different sites, all point towards a resolution of the *Oligobrachia* isotope and nutrition mystery. Specifically, it appears that that nutrition is thiotrophy based, and uptake of sediment inorganic carbon probably accounts for the observed highly negative carbon isotope signatures.

### Biogeography of symbionts and their hosts

We identified three symbiont types in samples of *O. webbi* (from Nyegga and HMMV) and *Oligobrachia* CPL-clade (from Storfjordrenna pingos and Bjørnøyrenna craters), BDJ, EFHI and A, of which, the former two were the most common. Both host species contained BDJ and EFHI symbionts which suggests that the two host species can establish a symbiosis with both of these symbiont types, however, the BDJ group was more often associated with *O. webbi* and the EFHI group was more often associated with the CPL-clade. *O. webbi* covers a large depth gradient  (~ 270 m to 1200 m water depth), whereas the CPL-clade has only been seen at shelf sites at less than 400 m water depth. Species replacements among animals have been observed to occur along depth gradients, even among seep species [13, 15, 32, 39, 52, 59, 60, 66]. This trend has not been studied widely among sediment bacteria, but there is some evidence to suggest that they too, can differ in species composition across water depth gradients [9]. Frenulate larvae are aposymbiotic and take up symbionts from the surrounding sediment [33, 49]. Water depth therefore represents a factor that potentially contributes towards the local bacteria available for high latitude seep *Oligobrachia* species to acquire. Differences in dominant symbiont types between the two host species may reflect depth driven differences in sediment bacterial communities. Type A was only found in the CPL-clade and since the CPL-clade has to date only been recovered from shelf locations, this bacterial group might also be restricted to shallower, shelf regions.

Finer scale local differences in available bacterial pools could explain some visible trends to symbiont communities in the two host species. For example, among *O. webbi,* it appears that there could be some degree of site-based differences to symbionts hosted. Nyegga individuals only contained symbionts from the BDJ type, whereas HMMV individuals contained symbionts from both the BDJ and the EFHI types. Within the CPL clade, there does not appear to be any site-based differences, such that samples from both the pingo and crater sites hosted both the BDJ and EFHI types (A was present in only a single sample). Furthermore, the comparison of our sequences with the ones from Lee et al. [46] obtained from Beaufort Sea CPL-clade members (420 m depth) showed that they belonged to our group F (note that Lee et al. [46] examined the V4-V5 region, so overlap between our ASVs and theirs is only for the V4 region). CPL-clade members therefore host similar symbiont strains or groups of strains regardless of which side of the Arctic they are located. Therefore, the site-based differentiation that appears to exist for *O. webbi*, albeit on a small number of specimens, does not appear to apply as much to the CPL-clade. Nonetheless, finer level grouping can be discerned with respect to specific collections; the samples from the C3 collection, for example, grouped tightly together (Fig. 4), which suggests that there could be small scale spatial structure to symbiont populations and uptake within individual sites.

Note that all the *O. webbi* samples (from Nyegga and HMMV) were frozen whereas all the CPL-clade samples were preserved in ethanol. Differences in community composition between the two host species could therefore potentially be due to different preservation techniques. However, the bacterial communities in the frozen Nyegga samples were highly distinct from those in the other frozen samples, from HMMV and, the HMMV samples were more similar to the ethanol preserved CPL-clade samples than to the frozen Nyegga samples (Figs. 3 and 4). Furthermore, among the frozen samples, differences in dominant bacterial groups were seen between individuals from the same location: group B was dominant in N1, but group C was dominant in the two replicates of the other individual from Nyegga, N2. Similarly, HM3 from HMMV was dominated by group I, however, the other samples from HMMV (HM2 and the two replicates of HM1) contained group J as the dominant group. Together, this suggests that the patterns we observed, with respect to both overall community structure and dominant bacterial groups among our samples, is not due to preservation method alone, and even though the effect of different fixation techniques cannot be completely discounted, it is likely that the patterns we observed are reflective of actual bacterial communities associated with these two species of *Oligobrachia.*

Opportunistically making use of the local sediment bacterial community instead of symbionts being strictly host-specific could contribute to the diversity of seep habitats (mud volcanoes, pockmarks, submarine slides, gas hydrate mounds and submarine canyons) that both *O. webbi* and *Oligobrachia* sp. CPL-clade inhabit [2, 11, 34, 35, 38, 45, 56, 57, 70, 72, 76]. *O. webbi* even inhabits non-seep habitats like fjords [75]. The large scale distributions of *O. webbi* and the CPL-clade is uncommon among frenulate siboglinid species [74, 81, 82]. Their ability to adjust their symbiosis based on local bacteria might have contributed towards them successfully colonizing such a wide range of seep and even non-seep habitats. High specificity with one or a limited number of bacterial strains might hinder their ability to survive across such diverse sites, because certain strains might be absent or present in low abundances at certain depths, latitudes, etc. Therefore, the strategy of being able to form symbiotic associations with multiple strains of bacteria could be key to the widespread distribution of *O. webbi* and *Oligobrachia* sp. CPL-clade across different kinds of north Atlantic and Arctic seeps, in contrast with the more limited distributions normally seen among frenulate siboglinid species.

Thiotrophic bacteria can use sulfide, thiosulfate or even elemental sulfur as their energy sources [1, 8, 27]. Hosting different types of bacteria, even if they are all thiotrophic might allow for *Oligobrachia* hosts to capitalize on more than one type of energy source, which could be yet another feature that makes these worms so adept at colonizing different kinds of seep and non-seep habitats. A common mechanism to avoid sulfide poisoning employed by animals living in high-sulfide environments such as vents and seeps is the partial oxidation of sulfide to the less toxic thiosulfate in animal tissues [7, 31]. It is not known whether and to what extent the studied *Oligobrachia* species can do this, but high, millimolar concentrations of sulfide have been measured in the porewater around both species studied here [34, 72, 73], and such circumstances might necessitate measures in addition to reliance on physiological adaptations (e.g., special hemoglobins, [51] to prevent sulfide toxicity, such as oxidation of sulfide to thiosulfate. Having bacterial partners that are capable of converting that thiosulfate into organic mass means that such activity and molecules do not go to waste [8].

### Amplification of non-symbiotic bacteria

It is of note that we obtained large numbers of sequences from symbiont-free samples. Some of those sequences could be due to contamination, for example, if trophosomes got ruptured during processing leading to symbiont-free tissue ending up with symbiotic bacteria. The closeness between host samples and trophosome samples supports this idea. Alternatively, worm body surfaces and/or tubes might contain significant populations of bacteria [61]. A number of frenulate siboglinids including *O. webbi* and the CPL-clade brood embryos and larvae in maternal tubes [49], Fig. 2E), and the adaptive significance of such behavior is not understood. A possible explanation could be exposure of the larvae to potential symbionts, if indeed, worm surfaces and tubes contain abundant populations of bacteria. This would allow for larvae, upon their release, to be proficient at recognizing strains in the surrounding environment that they can associate with, so that they can acquire them efficiently.

Regardless of whether the ASVs we obtained from symbiont-free tissue samples were from surface or tube bacteria as opposed to from the damaged trophosomes of those individuals, it highlights an important point about siboglinid associated bacteria. Care has to be taken when interpreting findings of bacterial sequences in samples: one should not assume that every sequence pertains to symbiotic bacteria. For this reason, we have been cautious in terms of considering sequences as belonging to symbiotic partners. ASVs abundant in trophosome tissue are the most likely to derive from symbionts, however, even this assumption is not fool-proof given that bacterial sequences can easily be amplified from symbiont-free tissue samples, as is evidenced from our results. Simply acquiring sequences from frenulate tissue does not imply that those sequences come from symbionts; our results even indicate that sequencing of trophosome tissue alone does not always allow us to differentiate between symbiotic and non-symbiotic bacteria. This needs to be kept in mind when interpreting past findings and future examinations of frenulate siboglinids.

## Conclusions

Our study reveals that high latitude seep *Oligobrachia* house thiotrophic bacterial symbionts, and that different host species can establish a symbiosis with the same bacterial strain. Other potential symbiont types, such as methanotrophs and heterotrophs do not appear to be part of the endosymbiont populations of these worms. The patterns of symbiont strains and host species and site associations, suggest that these two *Oligobrachia* species are opportunistic in their symbiosis, and develop their symbiotic associations with locally available bacteria. Based on patterns of distributions of host species and symbionts, we suggest that water depth contributes towards what constitutes the pool of locally available bacteria, though other factors need to be explored. High latitude *Oligobrachia* endosymbiosis is therefore intricate in ways not envisioned before, and this study represents a first step towards uncovering some of the aspects of a symbiosis that covers the circum-Arctic and the north Atlantic.

## Methods

### Sites and sample collections

*Oligobrachia* worms were collected from four different Arctic and sub-Arctic seep sites (Fig. 1). From south to north and east these are:

1. Nyegga pockmarks (64°N 5°E, 735 m water depth). On the northwestern flank of the large submarine Storegga slide offshore Norway, lies a series of pockmarks collectively referred to as the Nyegga site [37, 38]. Within the deepest of the pockmarks, pockmark G11, are a number of gas hydrate pingo features whose sediment surfaces are covered with bacterial mats and siboglinid worms (*O. webbi* and *Sclerolinum contortum*) [18, 37]. Samples of *O. webbi* were collected from one of the pingo features within G11 in May–June 2006, with blade cores using the remotely operated vehicle (ROV) *Victor 6000* during the VICKING cruise.
2. The Håkon Mosby mud volcano (HMMV; 72°N, 14°E, 1260 m water depth) is located in the northern Norwegian Sea and consists of an area where seafloor fluid and mud seepage follows a bull’s eye pattern with maximum discharge in a fauna-free central zone, surrounded by areas with more stable sediment but nonetheless high concentrations of sediment porewater sulfide and methane, inhabited by *O. webbi,* the moniliferan siboglinid, *Sclerolinum contortum* and bacterial mats [29, 65, 77]. Samples of *O. webbi* were collected with blade cores using the remotely operated vehicle (ROV) *Victor 6000* from dense worm tufts, during the same VICKING cruise when Nyegga was sampled (May–June, 2006).
3. Barents Sea pingo site (referred to as pingos or pingo site, 76°N, 15°E, 380 m water depth). This is an area in the central Barents Sea, within the Storfjord trough where a number of gas hydrate pingo features (or mounds) have been observed, with free gas emissions extending hundreds of meters above the summits of most pingos [35, 72, 76]. This site hosts a distinct, but morphologically cryptic *Oligobrachia* species from *O. webbi,* the species referred to as *Oligobrachia* sp. CPL-clade [70]. Samples were collected from the top of a pingo named GHP3, from among dense worm patches (e.g., Fig. 2A), in 2016 aboard the research vessel *Helmer Hanssen* (UiT The Arctic University of Norway). Collection number P1 was taken with a Van Veen grab directly operated from the ship and collection P2 was taken with a net scoop (two scooping events combined, see Fig. 1) with the ROV 30 K (Norwegian University of Science and Technology Centre for Autonomous Marine Operations and Systems)*.*
4. Barents Sea crater site (referred to as craters or crater site, 74°N, 27°E, 330 m water depth). This site consists of a large area of many squares of kilometers in the central Barents Sea, within the Bear Island (Bjørnøy) trough characterized by multiple craters and pingo-crater complexes, possibly created by blow-out explosions of subsurface methane hydrate reserves [2]. One pingo feature, the Yin Yang pingo-crater complex was examined in detail and observed to have sediment covered with siboglinid worms [72], specifically, also *Oligobrachia* sp. CPL-clade [70]. Samples were collected from the pingo of the Yin Yang complex in 2016 (Fig. 1), at the same time as sampling at the pingo site was conducted, with blade cores manipulated by the ROV 30 K.

For additional details and descriptions of these sites and their associated faunal communities, the reader is referred to the work cited in our descriptions above. The two species examined, *O. webbi* and *Oligobrachia* sp. CPL-clade (the latter is referred to often simply as the CPL-clade), represent the most widespread Arctic and sub-Arctic *Oligorachia* seep species, and together, are present at all active Arctic and sub-Arctic seep sites studied to date. However, they do not co-occur at any sites. Currently, one additional *Oligobrachia* species is known from high latitude seeps, an undescribed species, which has so far only been recovered from the Vestnesa pockmark seep site in the Fram Strait, where it co-occurs with *O. webbi* [74].

On both cruises during which the samples were collected (in 2006 and 2016), worms were removed from their tubes, placed on petri dishes containing filtered, chilled seawater and dissected under a microscope. Worms were divided into symbiont-free tissue (head, frenulum, zone of unpaired papillae, etc.) and symbiont-containing tissue (the trophosome). Therefore, samples consisted of either host-only (symbiont-free) tissue, or trophosome tissue, not both. The one exception was the N2 individual from Nyegga. The two samples from this individual consisted of a mix of both trophosome and symbiont-free tissue.

In most cases, a single sample was obtained from one individual worm. In cases where extraction out of the tube was particularly successful or the worm was particularly long, the worms was subdivided into two samples. Each individual sample was named with a letter or acronym representing the site from where it was collected (P for pingos, C for craters, HM for HMMV and N for Nyegga), followed by the collection number in the case of the pingo and crater samples (HMMV and Nyegga samples were all from a single location and collection event), and then a sequential number for the individual. For example, sample C3-6 represents a sample that is the 6th individual from collection number 3 from the crater site. Samples of symbiont-free tissues were appended with the suffix ‘h’ to refer to host-only tissue. When two samples were obtained from a single individual, each sample contained the additional suffix of ‘a’ or ‘b’ to represent that they were duplicates of the same tissue of a single individual. Despite these ‘duplicates’, each sample with its own name was treated as an individual sample, therefore even though, for example, P2-2 ha and P2-2hb are identical in that they are host-only tissue from the second individual from the P2 collection event, they still are different pieces of the same individual and were treated separately, and never pooled. Such ‘duplicate’ samples served as an internal control of the reproducibility of our sequencing analyses.

Tissue samples were preserved immediately upon extraction from the worms’ tubes in molecular grade absolute ethanol on board until analyses in the lab in the case of CPL-clade samples from the pingo and crater sites. *O. webbi* samples from HMMV and Nyegga were frozen at − 80 °C upon retrieval from their tubes, and kept frozen until labwork was conducted in 2017 along with the pingo and crater samples.

### DNA extraction and sequencing

All labwork was conducted at the Genomer platform of the Roscoff Marine Biological Station (France). DNA from all samples was extracted using the DNEasy Blood and Tissue kit (Qiagen, Germany) in accordance with the manufacturer’s instructions. The quality and quantity of obtained DNA were determined by 1% agarose gel electrophoresis and a spectrophotometer (NanoDrop).

A partial fragment of the 16S r RNA gene (V3-V4 region, 460 bp), amplified with primers indicated in the Illumina V3-V4 amplicon kit, and derived from Klindworth et al. [43], and dual indexing including a heterogeneity spacer [24], that increases diversity of the first bases read during sequencing.

PCR reactions were performed on a Gene-Amp PCR system 9700 thermocycler (Applied Biosystems) with a final volume of 25 µL using the following mix: 11.5 µL of extracted DNA was added to 12.5 µL 2 × KAPA Hifi HotStart Ready mix, and added to 1 µM of each of the tagged primers. Amplification involved an initial denaturation step at 95 °C for 3 min, followed by 25 cycles at 95 °C for 30 s, 55 °C for 30 s and 72 °C for 30 s, and followed by a final extension step at 72 °C for 5 min. PCR products were measured using fluorescence by Qubit (Thermofisher). All amplification products were run on a bioanalyzer (Agilent) to verify the size of the amplicons.

The samples were purified using calibrated Ampure XP beads. The purified PCR product was used to prepare a DNA library by following the Illumina 16S Metagenomic Sequencing Library preparation protocol. All PCR products were then pooled at equimolar concentrations. Paired-end sequencing of the amplicons was performed by the Genomer (Roscoff, France) Platform using MiSeq Illumina technology (2 × 300 bp).

### Bioinformatics processing and statistical analyses

Raw reads were demultiplexed, quality-checked, filtered and trimmed (including removing primer regions and heterogeneity spacers), paired ends were assembled, chimeric sequences were discarded, and reads were denoised using the DADA2 package in R to generate a list of Amplicon Sequence Variants (ASVs). General taxonomic affiliations were obtained and assigned to the individual ASVs using the Silva database version 138 [58] with the *assignTaxnomy* function in DADA2, using default bootstrap confidence on assignations.

An ASV abundance table was produced and singletons as well as ASVs classified as mitochondria, Archaea, Eukaryota, and unassigned at the kingdom level, were discarded. Since the goal of this study was to investigate symbiont communities, mitochondria, eukaryotes and unassigned sequences at the kingdom level were excluded since those sequences would not pertain to symbionts. Archaea are important members of the sediment microbial community at seeps, however, we obtained very few reads that were classified as Archaea (765 in total), and to date, no siboglinids have been found to harbor endosymbiotic Archaea [33, 87], which is why we further excluded sequences assigned to Archaea. The ASV table was then used for conducting community analyses with the vegan package in R [55] and Primer 7 [12]. Diversity indices and evenness were calculated to examine the diversity of the bacterial communities across the different samples. Raw numbers or reads of ASVs for each sample were normalized based on total reads per sample, and fourth root transformed to account for the very different numbers of reads obtained. The standardized and transformed ASV table was used to construct a Bray–Curtis similarity matrix which was then used for constructing dendrograms for cluster analysis.

Pairwise Kimura distances between sequences were calculated by with Mega X [84]. Pairwise distances were also calculated between sequences obtained in this study with symbionts from other siboglinid species from cold seeps.

### Transmission electron microscopy

The trophosomes of five individuals of *O. webbi* collected during the VICKING cruise from the Nyegga-Storegga site were fixed and processed for transmission electron microscopy. This involved fixing them for 4 h with 4% glutaraldehyde in a phosphate buffered saline (PBS) solution at sea, rinsing and storing them in filtered seawater with sodium azide (0.13 g for 50 mL) and then post fixing and dehydrating with sodium cacodylate and ethanol (details in [70]. A total of 28 grids were prepared from which 39 photographs were taken with a JEOL SX 1200 transmission electron microscope at the facility located in Roscoff Marine Station.

## Supplementary Information

**Additional file 1**. The complete table of ASVs used in this study (.xlx). Full sequences are in the first column. Each ASV is given a sequential number (ASV number) written in the second column. Numbers in the rest of the table represent the number of reads obtained for the sequence in each sample. The last columns are the Silva based classifications of each ASV. Fastq files will be deposited in GenBank.

## Data Availability

The datasets supporting the conclusions of this article are included within the article (and its additional files). Additionally, fastq files are uploaded to GenBank.
